# Light-Mediated Inhibition of Colonic Smooth Muscle Constriction and Colonic Motility *via* Opsin 3

**DOI:** 10.3389/fphys.2021.744294

**Published:** 2021-12-16

**Authors:** William Dan, Ga Hyun Park, Shruti Vemaraju, Amy D. Wu, Kristina Perez, Meenakshi Rao, Dan E. Berkowitz, Richard A. Lang, Peter D. Yim

**Affiliations:** ^1^Department of Anesthesiology, Vagelos College of Physicians and Surgeons, Columbia University, New York, NY, United States; ^2^The Visual Systems Group, Abrahamson Pediatric Eye Institute, Cincinnati Children’s Hospital Medical Center, Cincinnati, OH, United States; ^3^Division of Pediatric Ophthalmology, Center for Chronobiology, Cincinnati Children’s Hospital Medical Center, Cincinnati, OH, United States; ^4^Department of Pediatrics, Boston Children’s Hospital, Harvard Medical School, Boston, MA, United States; ^5^Department of Anesthesiology and Perioperative Medicine, School of Medicine, University of Alabama, Birmingham, AL, United States; ^6^Division of Developmental Biology, Cincinnati Children’s Hospital Medical Center, Cincinnati, OH, United States; ^7^Department of Ophthalmology, College of Medicine, University of Cincinnati, Cincinnati, OH, United States

**Keywords:** colon, motility, opsin, relaxation, neuron

## Abstract

Opsin photoreceptors outside of the central nervous system have been shown to mediate smooth muscle photorelaxation in several organs. We hypothesized that opsin receptor activation in the colon would have a similar effect and influence colonic motility. We detected Opsin 3 (OPN3) protein expression in the colonic wall and demonstrated that OPN3 was present in enteric neurons in the muscularis propria of the murine colon. Precontracted murine colon segments demonstrated blue light (BL) -mediated relaxation *ex vivo*. This photorelaxation was wavelength specific and was increased with the administration of the chromophore 9*-cis* retinal and a G protein receptor kinase 2 (GRK2) inhibitor. Light-mediated relaxation of the colon was not inhibited by L-NAME or tetrodotoxin (TTX). Furthermore, BL exposure in the presence of 9*-cis* retinal decreased the frequency of colonic migrating motor complexes (CMMC) in spontaneously contracting mouse colons *ex vivo*. These results demonstrate for the first time a receptor-mediated photorelaxation of colonic smooth muscle and implicate opsins as possible new targets in the treatment of spasmodic gastrointestinal dysmotility.

## Introduction

Irritable bowel syndrome (IBS) is a chronic gastrointestinal disorder with a multifactorial etiology and is characterized by abdominal pain, bloating, and altered stool patterns (Ohman and Simrén, 2010). Historically, therapy has focused on the management of symptoms; for diarrhea-predominant IBS (IBS-D), antispasmodics are used to treat hypermotility and cramps (Chang et al., 2014). With high rates of prevalence and psychological comorbidity, IBS poses a significant health care burden (Spiegel, 2009; Lovell and Ford, 2012; Geng et al., 2018). While activation of the G protein-coupled β_3_-adrenoceptor has been shown to mediate intestinal smooth muscle relaxation *ex vivo* (Roberts et al., 1999), such treatment did not produce changes in human gastrointestinal or colonic transit (Grudell et al., 2008). There remains a demand for fast-acting, easily reversible antispasmodics.

In recent decades, taste, visual, and olfactory receptors have been discovered in non-sensory organs (Dalesio et al., 2018). Moreover, these ectopic sensory G protein-coupled receptors (GPCRs) have been shown to function in physiologic and pathophysiologic processes, thus presenting as a class of potential therapeutic targets for treatment of human diseases. Opsins are G protein-coupled, light-sensitive receptors responsible for vision and have been discovered outside the central nervous system with expression in multiple organs including smooth muscle, skin, white blood cells, and liver (Sikka et al., 2014; Barreto Ortiz et al., 2018). The endogenous non-visual opsin subtypes are Opsin 3 (OPN3, panopsin), Opsin 4 (OPN4, melanopsin), and Opsin 5 (neuropsin; Terakita, 2005). Our group recently demonstrated the expression and functional role of OPN3 in airway and uterine smooth muscle relaxation (Yim et al., 2019, 2020). We demonstrated blue light (BL)-induced relaxation of acetylcholine (Ach)-precontracted airway smooth muscle. In *ex vivo* preparations of uterus smooth muscle from full-term pregnant humans, BL significantly attenuated oxytocin-induced contractile tension and frequency. In the present study, we questioned whether blue light would have similar effects on colonic smooth muscle.

Mammalian opsins are a unique class of 7-transmembrane GPCRs, becoming light sensitive upon covalent binding of a carotenoid analog. Photon incidence isomerizes the chromophore and causes a conformational change in the opsin protein, leading to phototransduction (Wald, 1968). While 11*-cis* retinaldehyde is the endogenous ligand that binds to the chromophore pocket of the opsin apoprotein in the human eye, 9*-cis* retinaldehyde (9*-cis* retinal) is commonly used in *ex vivo* and *in vitro* opsin studies due to its preserved ability to form stable, photosensitive pigments (Hubbard and Wald, 1952; Pepperberg et al., 1978; Corson et al., 1990; Yim et al., 2019, 2020). The phenomena of blue light-mediated photorelaxation observed in airway and uterine smooth muscle were enhanced by dark adaptation, pretreatment with the known opsin chromophore 9*-cis* retinal, and inhibition of G protein receptor kinase 2 (GRK2), a kinase well known to phosphorylate and inactivate a diverse number of GPCRs. In previous studies by our group and others of opsin receptor-mediated smooth muscle photorelaxation, it is hypothesized that GRK2 (also known as β-adrenoceptor kinase) mediates opsin desensitization by facilitating β-arrestin binding to the GPCR (Shichi and Somers, 1978; Moore et al., 2007; Barreto Ortiz et al., 2018; Yim et al., 2019, 2020). Inhibition of GRK2 phosphorylation of the opsin receptor would limit light-induced inactivation and internalization of the receptor due to ambient light exposure, maximizing the observed photorelaxation responses.

We report the novel expression of OPN3 in the walls of mouse colon within neuronal cells and cells within the smooth muscle layer. We implicate OPN3 as a mediator of colonic photorelaxation by demonstrating that blue light combined with chromophore administration and GRK2 inhibition attenuates carbachol-induced colonic constrictions in organ bath and spontaneous peristaltic contractions in *ex vivo* murine whole colon preparations.

## Materials and Methods

### Animals

#### Imaging Studies

All experiments were approved by Cincinnati Children’s Hospital Medical Center Institutional Animal Care and Use Committee and were in accordance with National Institute of Health guidelines. Mice were anesthetized under isoflurane and killed by cervical dislocation.

#### Functional Colon Studies

Mouse protocols were approved by the Columbia University Institutional Animal Care and Use Committee. About 16–20-week-old male C57BL/6 J mice (Jackson Laboratory, Bay Harbor, ME) were euthanized with intraperitoneal injections of sodium pentobarbital (40 mg/kg).

### Opsin Expression in Murine Colon

Proximal colon was harvested and flushed with ice cold PBS prior to fixation in 4% PFA at 4°C for 18 h. After washing in PBS and cryoprotection in PBS containing 30% sucrose, colon samples were embedded in OCT (Tissue Plus, Fisher Scientific 23-730-571) and cryosectioned at 16 μm. Sections were washed in PBST (0.3% Triton X-100) and blocked in 3% normal donkey serum PBST for 2 h at room temperature. Sections were incubated in primary antibodies (1:500) to GFP (ab13970,), β-tubulin III (Biolegend 802001), or α-SMA (Sigma A2547) prepared in blocking buffer at 4°C for 18 h. Following three 20-min washes in PBST, sections were incubated in fluorescent-conjugated secondary antibodies (1:800) purchased from Jackson ImmunoResearch for 1 h at room temperature along with Hoechst 33342 for cell nuclei visualization. After washing with PBST, the sections were coverslipped with Fluoro-gel (EMS). Tissues from Tg(Opn3-eGFP)JY3Gsat (MMRRC stock number 030727-UCD) and GFP-negative control C57BL/6 mice embedded in the same OCT block and processed in parallel. Images were captured with Nikon Eclipse Ti2 confocal microscope.

### Myography

Freshly harvested murine colons were flushed with modified Krebs–Henseleit (KH) Buffer (in mM: NaCl 137, KCl 2.9, CaCl_2_ 1.8, MgCl_2_ 2.1, NaH_2_PO_4_ 0.4, NaHCO_3_ 11.9, D-Glucose 5.6, pH 7.4). Transverse, annular segments of distal colon were excised ≥1 cm from the anus under a dissecting microscope. Colon rings were mounted in chambers of a myograph (DMT 620M Multi Wire Myograph, Danish Myo Technology, Ann Arbor, MI). The rings were equilibrated in KH buffer at 37°C, bubbled at 95% O_2_/5% CO_2_ and resting tension adjusted to 1.0 g for 1 h. To assess tissue viability, colonic rings were isometrically contracted with 3 cycles of increasing log concentrations of ACh (10 nM–1 mM; Sigma-Aldrich, St. Louis, MO).

**Figure 1:**
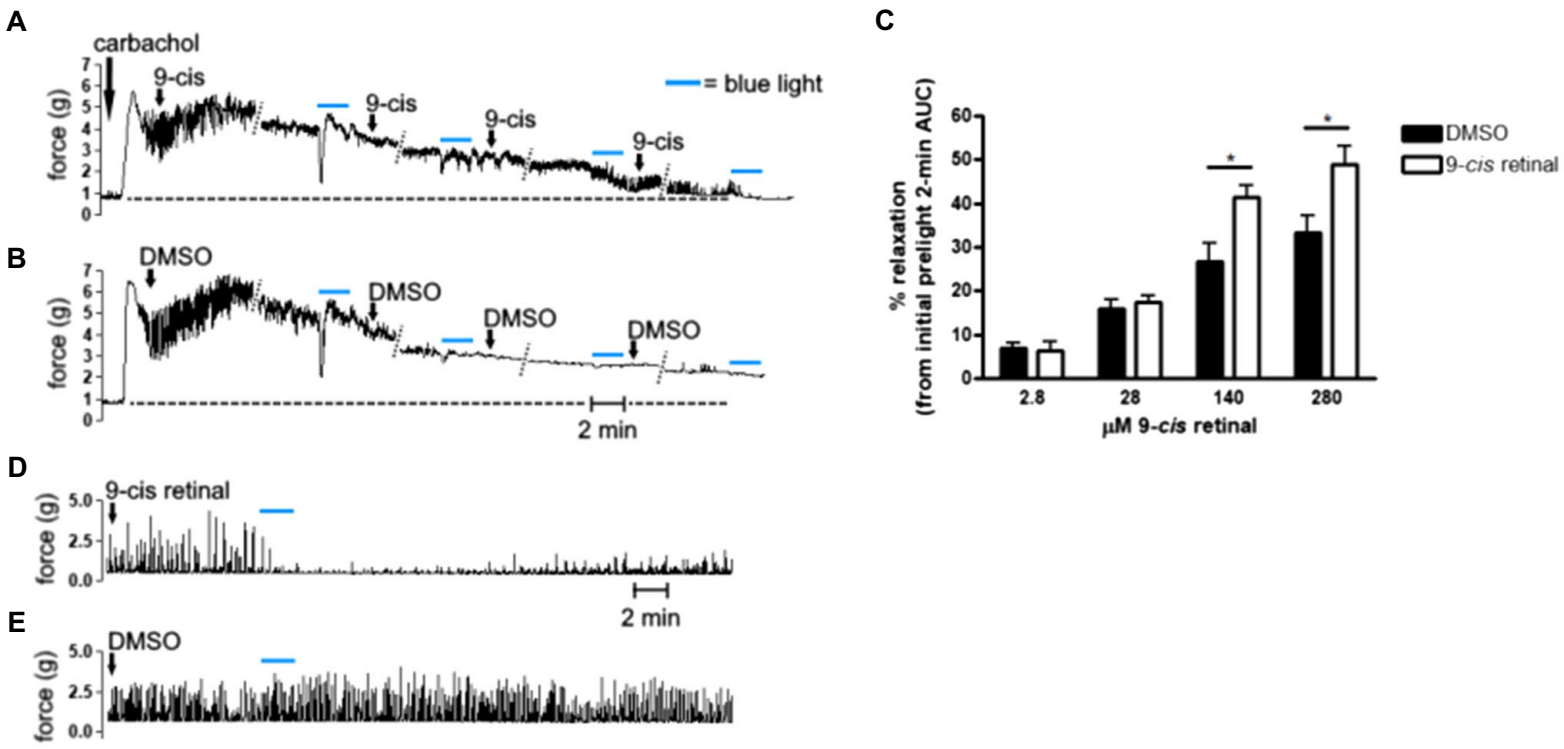
**(A,B,D,E)** Representative muscle force tracings of *ex vivo* murine colon rings pretreated with tetrodotoxin (TTX) 1 μM and contracted with carbachol 100 μM in wire myography experiments. **(A,B)** Force tracings of *ex vivo* colon ring administered increasing final concentrations of **(A)** 9-*cis* retinal (9-*cis*; 2.8, 28, 140, and 280 μM), or **(B)** DMSO vehicle with 15 min in between doses. About 12 min after each dose, rings were exposed to blue light (BL; 435 nm λ) for 2 min as indicated by the blue bars. Diagonal dotted lines indicate tracing sections cropped for brevity. Dashed line extending perpendicularly from the y-axes approximates baseline, precarbachol tension and demonstrates that 9-cis + BL-treated rings **(A)** approached baseline tension sooner than DMSO + blue light-treated rings. **(C)** Area under the curve (AUC) analysis of precontracted *ex vivo* colon rings administered increasing doses of 9-*cis* retinal or DMSO vehicle, with intervening blue light treatments. About 2-min AUC during blue light exposures were compared to the 2-min AUC immediately preceding the first light treatment (initial prelight 2-min AUC) to calculate % relaxation. Blue light combined with doses of 9-*cis* retinal at 140 and 280 μM produced significantly more relaxation than the corresponding administrations of DMSO and blue light (^*^p < 0.05, *n* = 11–26 rings per group). **(D,E)** Separate *ex vivo* myography experiments in which precontracted murine colon rings were administered either **(D)** 280 μM 9-*cis* retinal or **(E)** DMSO vehicle and subsequently exposed to blue light for 2 min. Tracing in **(D)** demonstrates recovery of carbachol-induced contractions approximately 20–30 min after cessation of blue light.

**Figure 2:**
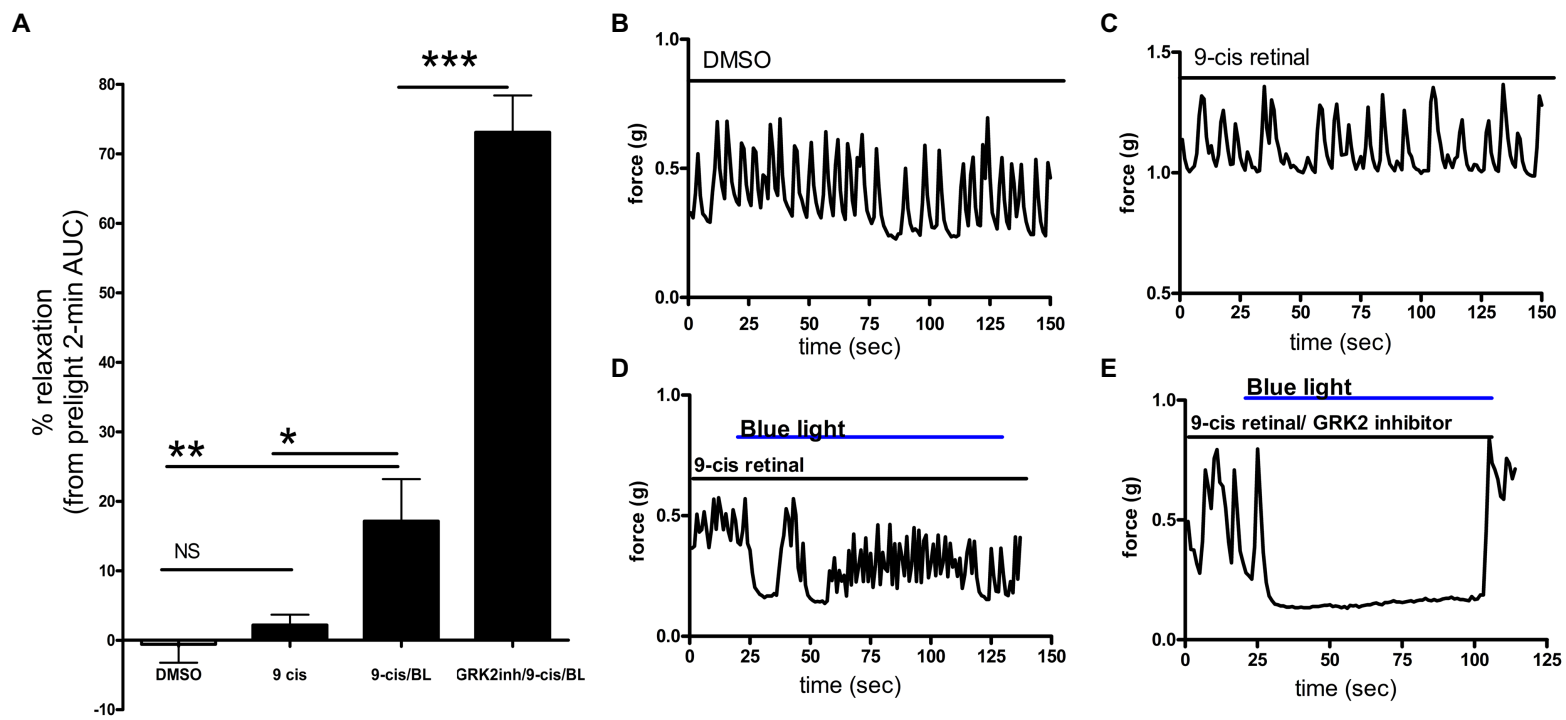
**(A)** Area under the curve analysis of *ex vivo* murine colon rings precontracted with carbachol with pretreatment with DMSO, 9*-cis* retinal or 9*-cis* retinal + G protein receptor kinase 2 (GRK2) inhibitor followed by no light or BL treatment. Rings treated with 9*-cis* retinal and blue light exhibited significantly more relaxation (17 ± 6.0%) vs. rings only pretreated with 9*-cis* (2.2 ± 1.5%) and vehicle-pretreated rings not treated with light (^*^p < 0.05, ^**^p < 0.01, *n* = 8–12 rings per group). Colonic rings pretreated with a GRK2 inhibitor + 9*-cis* retinal followed by blue light exposure demonstrated significantly greater photorelaxation (73 ± 5.3%) compared to rings pretreated with 9*-cis* retinal alone and blue light. (^***^p < 0.001, *n* = 8–15 rings per group). **(B–E)** Representative muscle force tracings of *ex vivo* murine colon rings pretreated with TTX 1 μM and contracted with carbachol 100 μM in wire myography experiments. Colon segments were pretreated with 0.1% DMSO vehicle, 9*-cis* retinal 28 μM, or 9*-cis* retinal + GRK-2 inhibitor (20 μM) and exposed to blue light (435 nm λ). Solid bars indicate the duration of the treatment.

Following the final ACh dose response and three KH buffer changes, the rings were returned to a resting tension of 1.0 g, and the room was darkened for 20 min. Rings were pretreated with or without (+/−) 9*-cis* retinal 2.8–280 μM (Sigma-Aldrich, St. Louis, MO, United States). Around 28 μM 9-*cis* retinal was selected in some experiments as this was the concentration which produced significant photorelaxation vs. no 9-*cis* retinal in a previous study (Yim et al., 2019). In some experiments, rings were treated with +/− GRK2 inhibitor {Methyl 5-[(E)-2-(5-nitrofuran-2-yl)ethenyl]furan-2-carboxylate, Santa Cruz Biotechnology, Dallas, TX, United States} 20 μM. We previously achieved maximal response with 200 μM GRK2 inhibitor and thus used an order of magnitude lower dose in the present study to ensure a submaximal effect. We also inhibited tetrodotoxin (TTX)-sensitive neuronal effects by pretreating all tissues with the Na^+^ channel blocker TTX at 1 μM (EMD Millipore, Jaffrey, NH, United States). In some experiments, we also pretreated tissues with 100 μM of N(ω)-nitro-L-arginine methyl ester, L-NAME (Sigma-Aldrich, St. Louis, MO, United States), to inhibit nitric oxide (NO) synthase activity. In all myography experiments except those used for data in Figure 1, 9-*cis* retinal and GRK2 inhibitor were administered at the same time as TTX. In myography experiments used for Figure 2 experiments, 9-*cis* retinal was initially co-administered with carbachol. All pretreatments (9-cis retinal, GRK2 inhibitor, TTX, and L-NAME) were performed 1 h prior to light treatment, except for 9-cis dose response (Figure 1), which were 15 min pretreatments.

After 20 min of dark and pharmacologic pretreatment, the rings were isometrically contracted with the cholinergic agonist carbachol 1 or 100 μM (Sigma-Aldrich, St. Louis, MO, United States) or 80 mM KCl. Around 1 μM carbachol was used to produce similar contractile tension (EC_50_) as 80 mM KCl. Following the establishment of stable contractions, colon rings were exposed to blue (435 nm λ) or red (635 nm λ) light at 5 mW intensity from 5 cm distance (PE4000, CoolLED, Andover, England), or no light. Myograph analog signals were amplified with a BIOPAC MP100 and analyzed using AcqKnowledge software (version 3.9.1) as muscle tension expressed in grams. Area under the curve (AUC) analyses were conducted by comparing the force–time integral of the 2-min duration of light or no light treatment to the force–time integral of the 2-min duration immediately preceding the first light treatment of the same colonic ring.

In resting tension experiments, we pretreated colonic smooth muscle with GRK2 inhibitor {Methyl 5-[(E)-2-(5-nitrofuran-2-yl)ethenyl]furan-2-carboxylate} and 9-cis retinal as described above including all wash steps and precontractions (acetylcholine dose responses). Before and after 100 μM carbachol treatment, colonic rings were treated with 435 nm blue light for 2 min to demonstrate relaxation at basal tension and to confirm the photorelaxation response remained intact.

### Colonic Migrating Motor Complexes

Oxygenated KH Buffer (formula above) was flushed through tubing attached to a syringe of a two-chambered organ bath setup. A custom designed 3D-printed organ bath was next placed on top of a heating plate controlled by a thermostat, maintaining the bath temperature between 34.5 and 35°C. The chamber was filled and constantly circulated with 200 ml KH buffer, which was bubbled with 95% O_2_/5% CO_2_, and the serosal compartment was superfused at a flow rate of 5 ml/min. The entire colon was excised and mounted under red light; the proximal and distal ends were cannulated by the tubing and secured using surgical suture. The proximal end was flushed and intraluminal pressure maintained by a 3 cc column of KH buffer. The length of the colon was recorded by measuring the distance between the proximal and distal cannulations. A video camera was mounted 10–15 cm above the organ bath. About 28 μM of 9*-cis*-retinal was added to the circulating bath and the colon allowed to equilibrate for 30 min. About 2 × 15 min videos of intestinal movement were recorded under red (660 nm λ) light in order to measure baseline motility parameters (baseline). The preparation was then recorded for an additional 2 × 15 min under either red light (ctrl, time decay) or under blue (435 nm λ) light and compared to baseline.

Colon edges were continuously detected from video recordings using custom-written edge detection software to create a summary file, from which a spatiotemporal map was then generated using MATLAB (R2019a; Welch et al., 2014; Swaminathan et al., 2016; Rao et al., 2017). These spatiotemporal maps depict colon widths (color spectrum) at different colon widths at different points along the length of the colon (x-axis) over time (y-axis). Colonic contractions traveling more than half the length of the tissue were considered as colonic migrating motor complexes (CMMCs). CMMC frequency, speed, length, and duration were quantified.

### Statistical Analysis

Groupwise comparison was analyzed by one-way ANOVA with Bonferroni *post hoc* test using Prism 4.0 (GraphPad, San Diego, CA, United States). Student’s *t*-test was used for two group comparisons. Statistical significance was established at *α* = 0.05 and values are expressed as mean ± SEM.

## Results

### Evaluation of Opsin 3 Protein Expression

**Figure 3:**
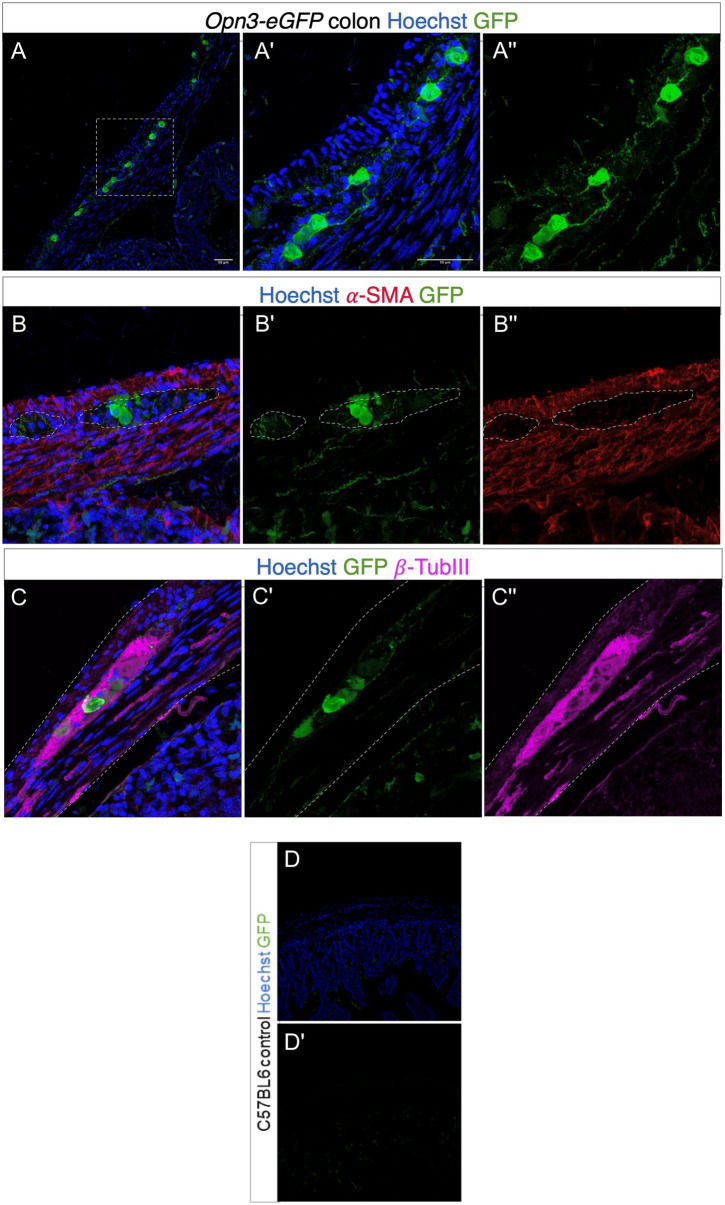
Expression of Opsin 3 (Opn3)-eGFP in adult mouse proximal colon. **(A–C)** Opn3-eGFP expression in neurons within the myenteric plexus demonstrated by green fluorescent staining. Red fluorescent staining demonstrates positively labeled α-SMA. Violet fluorescent staining demonstrates positively labeled β-Tubulin III. Blue fluorescence indicates positive Hoechst nuclear staining. **(A',A'')** Magnified images of area demarcated in **(A)**. **(B,B')** Opn3-eGFP labeling shows discrete low-level expression inside the smooth muscle layer (labeled with α-SMA). **(C,C')** Opn3- eGFP labeling is evident in neuronal cells (labeled with β-Tubulin III). Note GFP+ nerve fibers in **(A,B)**. Gray dashed lines in **(B,C)** indicate areas of interest. Lighter staining as well as discrete cellular areas in the smooth muscle layers is not specifically colocalized within SMA-positive cells, but is in the muscle layer appreciated in **(A'')** as well as (B'). Bottom panel (control; **D,D'**) Proximal colon from C57BL6 animals that have no Opn3-eGFP expression. Note endogenous background fluorescence within crypts but none within the colon muscle/neuron layer.

Using a mouse genetically modified to express Opsin 3 coupled to eGFP, fusion protein, we found high levels of Opsin 3 reporter expression in specific cells in between the outer longitudinal and inner circular muscle layers of the colon (Figure 3). GFP-expressing cells were immunoreactive for the pan-neuronal marker TUBB3, confirming that Opsin 3 is expressed within a subset of myenteric neurons. Furthermore, low level of expression invades into the smooth muscle layers but does not specifically costain with alpha-SMA staining. GFP-negative mice were used as controls visualized under identical optical conditions.

### 9*-cis* Retinal-Treated Murine Colonic Tissue Relaxes With Blue 435 nm λ Light

**Figure 4:**
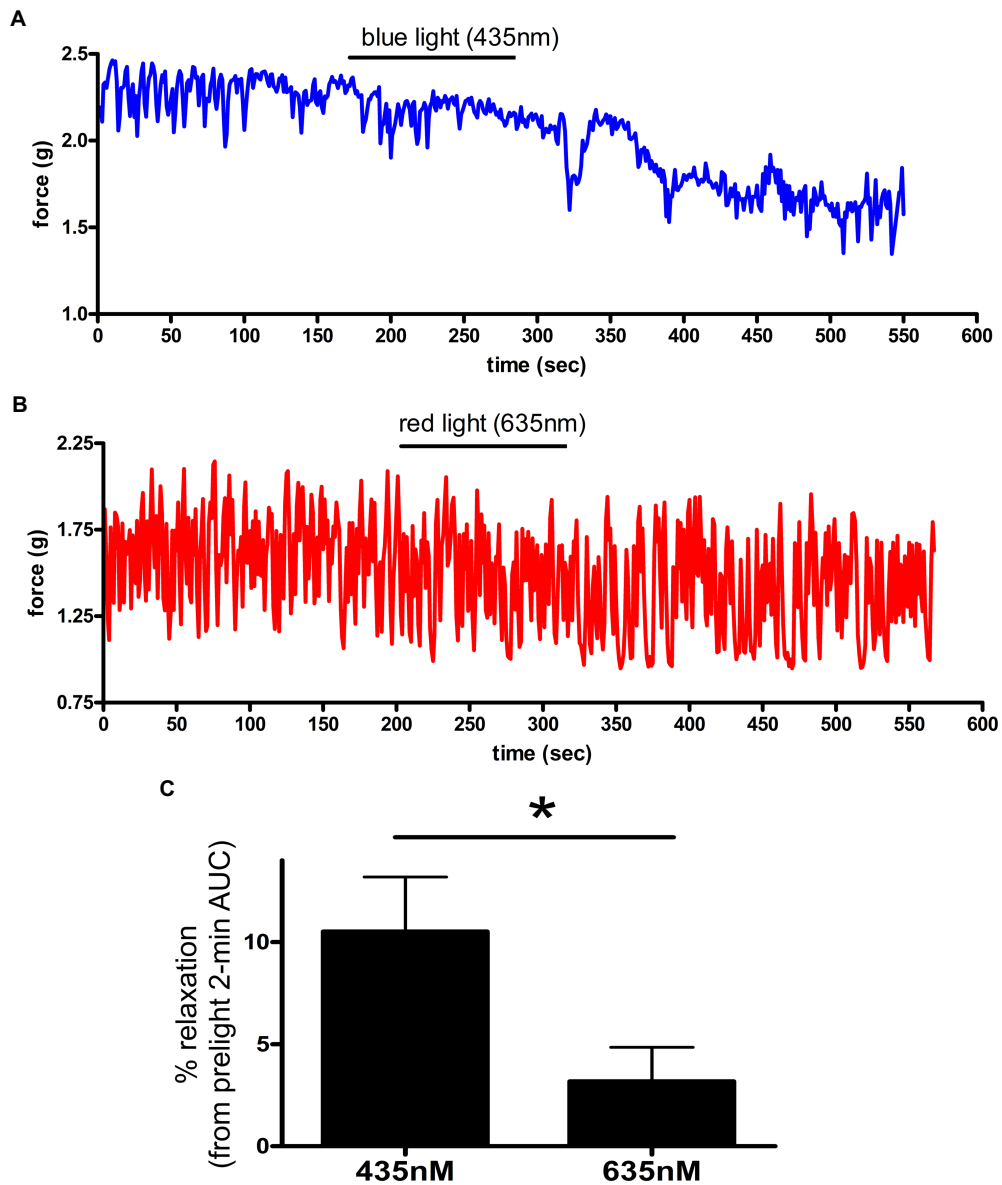
**(A,B)** Representative muscle force tracings of *ex vivo* murine colon rings pretreated with TTX 1 μM/9*-cis* retinal 28 μM and contracted with carbachol 100 μM in wire myography experiments. Blue tracing **(A)** represents a colonic ring exposed to blue light (435 nm) indicated by the black bar. Colon ring represented by red tracing **(B)** was treated with 635 nm λ light as a negative control (duration indicated by the black bars). **(C)** AUC analysis of relaxation of *ex vivo* murine colon rings precontracted with carbachol and TTX/9-*cis* retinal pretreatment. Rings exposed to 435 nm λ light demonstrated a 10 ± 2.7% relaxation, while the 635 nm λ light group demonstrated a 3.2 ± 1.7% relaxation (mean ± SEM, ^*^p < 0.05, *n* = 5–6 rings).

The unique combination of chromophore (exogenously added 9*-cis* retinal) and opsin receptor produces a photosensitive complex that is wavelength specific (Figure 4). To determine whether murine colonic rings were specifically sensitive to blue light (435 nm λ light), we measured relaxation of carbachol precontracted *ex vivo* murine colonic rings during blue vs. red light exposure. About 435 nm λ blue light induced a 10.5 ± 2.7% relaxation of the initial carbachol contraction, while 635 nm λ red light induced significantly less relaxation of 3.2 ± 1.7% (mean ± SEM, *p* < 0.05, *n* = 5–6 rings).

### 9-*cis* Retinal Dose-Dependent Enhancement of Blue Light Photorelaxation

Increasing concentrations of 9-*cis* retinal were administered to carbachol precontracted murine colonic rings with intervening exposures to blue light (Figure 1). Light-mediated relaxation after 140 and 280 μM treatments of 9-*cis* retinal was significantly greater than corresponding treatments of vehicle and blue light in control rings (*p* < 0.05). Figure 2D demonstrates that colonic rings pretreated with 280 μM 9-*cis* retinal maintained viability and qualitatively resumed carbachol-induced contractions after blue light-mediated photorelaxation.

### Inhibition of GRK Enhances Blue Light Photorelaxation

To determine whether photorelaxation of *ex vivo* murine colonic rings desensitizes through a GPCR-mediated pathway, we pretreated dark-adapted colonic rings with the known opsin chromophore 9*-cis* retinal and GRK2 inhibitor, a kinase well characterized in heterologous phosphorylation and desensitization of diverse G protein-coupled receptors. Murine colonic rings were pretreated with 9*-cis* retinal or DMSO vehicle and then with or without GRK2 inhibitor, followed by contraction with carbachol and exposure to blue light (Figure 2). 9*-cis* retinal pretreated rings demonstrated relaxation of colonic tissue in response to blue light of 17.1 ± 6.0% of the carbachol contraction (mean ± SEM, *n* = 15), while rings also pretreated with the GRK2 inhibitor demonstrated a significantly augmented relaxation to blue light; 73.1 ± 5.3% of the initial carbachol contraction (mean ± SEM, *n* = 10; *p* < 0.001 when comparing GRK2 inhibitor/9*-cis* retinal vs. 9*-cis* retinal pretreatment alone). Vehicle (0.1% DMSO) pretreated rings and 9*-cis* pretreated rings in the absence of blue light demonstrated minimal changes in contractile tone −0.6 ± 2.6%, (mean ± SEM, *n* = 10), and 2.2 ± 1.5%, (mean ± SEM, *n* = 12), respectively. There was a significant difference between 9*-cis* pretreatment and 9*-cis* pretreatment with blue light (*p* < 0.05). There was no significant difference between vehicle control and 9*-cis* pretreatment in the absence of blue light.

### Colonic Photorelaxation Is Independent of Nitric Oxide Synthase and Tetrodotoxin-Sensitive Nerves

**Figure 5:**
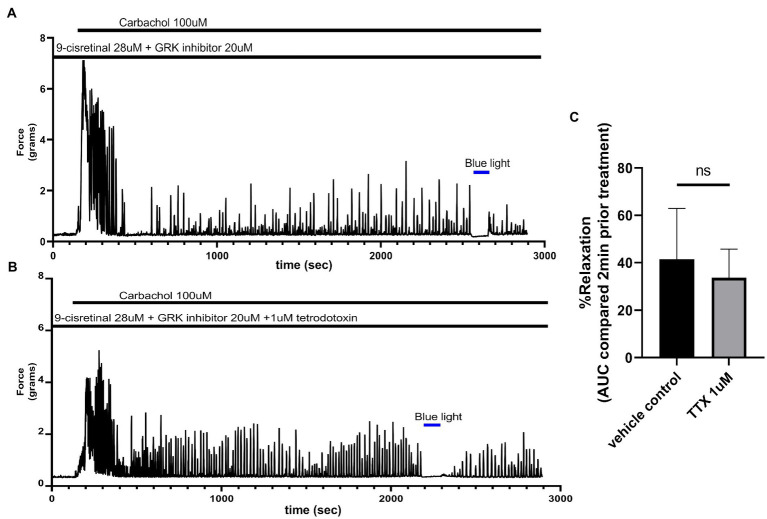
**(A)** Representative force tracing of colonic rings pretreated with 9-*cis* retinal 28 μM + GRK2 inhibitor 20 μM and constricted with 100 μM carbachol. Pretreatments depicted by the black bars. Blue bar depicts a 2 min 435 nm blue light treatment. **(B)** Representative force tracing of colonic rings pretreated with 9-*cis* retinal 28 μM + GRK2 inhibitor 20 μM + 1 μM tetrodotoxin and constricted with 10 μM of carbachol. Pretreatments are depicted by the black bars, and the blue bar depicts a 2 min 435 nm blue light treatment. **(C)** Area under the curve analysis of blue light-induced photorelaxation of rings precontracted with or without 1 μM TTX. There was no significant difference between the means of TTX and vehicle treatment (42 vs. 34%, respectively), *n* = 4.

To determine whether TTX could inhibit the relaxation associated with 435 nm blue light treatments, we exposed colonic rings in organ bath *ex vivo* experiments to 1 μM TTX or buffer along with 28 μM of 9-cis retinal and 20 μM of GRK2 inhibitor {Methyl 5-[(E)-2-(5-nitrofuran-2-yl)ethenyl]furan-2-carboxylate} prior to 100 μM carbachol. In the buffer pretreatment group, 435 nm light treatment demonstrated a 41.5 ± 10.7% (mean ± SEM, *n* = 4), compared to the 1 μM TTX pretreatment group which demonstrated a 33.8 ± 11.9% (mean ± SEM, *n* = 4), during 435 nm blue light treatment. There was not a statistical difference between these two groups (Figure 5).

**Figure 6:**
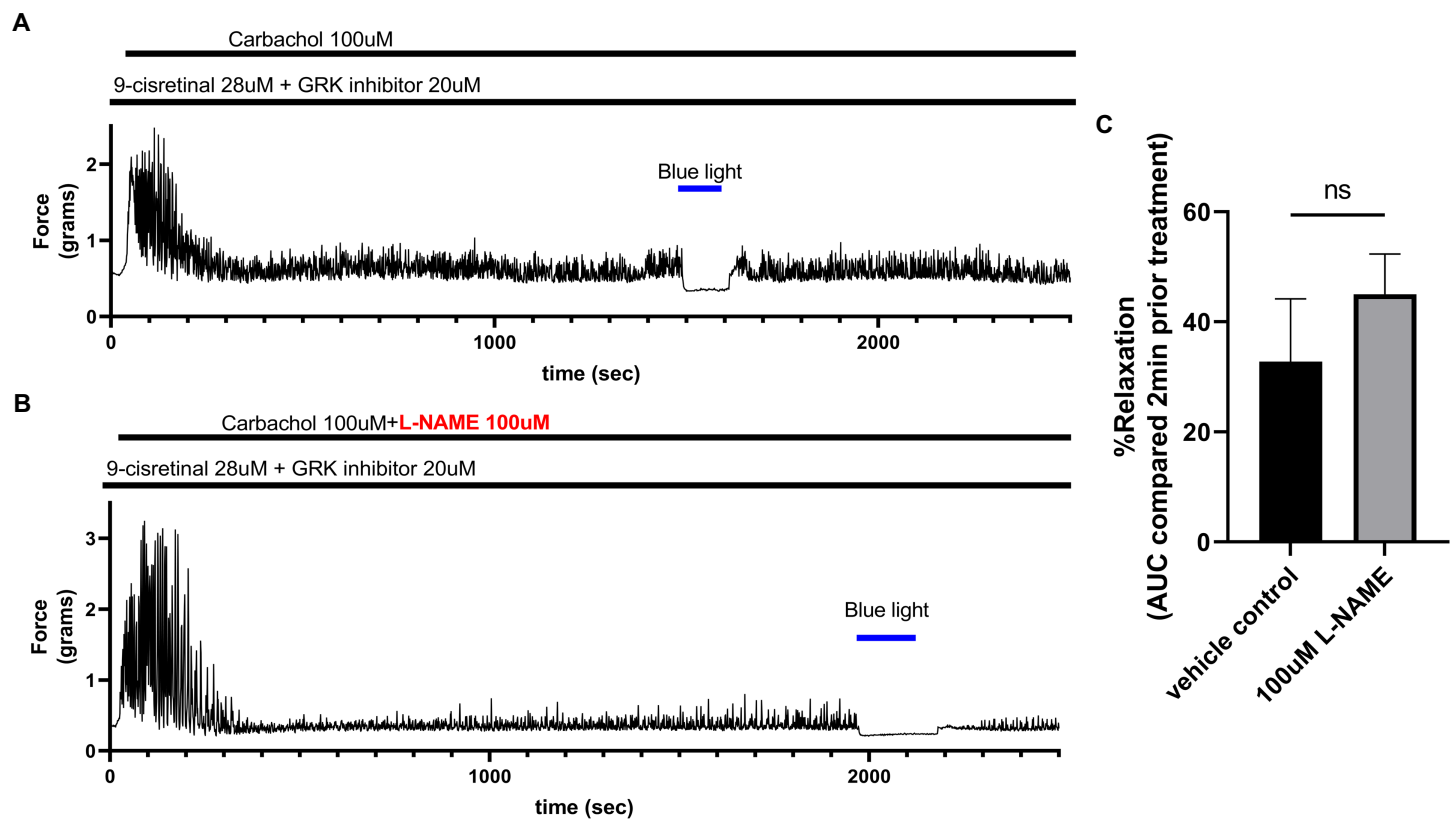
**(A)** Representative force tracing of colonic rings pretreated with 9-*cis* retinal 28 μM + GRK2 inhibitor 20 μM and constricted with 100 μM carbachol. Pretreatments depicted by the black bars. Blue bar depicts a 2 min 435 nm blue light treatment. **(B)** Representative force tracing of colonic rings pretreated with 9-*cis* retinal 28 μM + GRK2 inhibitor 20 μM + 100 μM L-NAME and constricted with 10 μM of carbachol. Pretreatments are depicted by the black bars, and the blue bar depicts a 2 min 435 nm blue light treatment. **(C)** Area under the curve analysis of blue light-induced photorelaxation of rings precontracted with or without 100 μM L-NAME. There was no significant difference between the means of light-induced relaxation with L-NAME vs. vehicle pretreatment (45 vs. 33%, respectively), *n* = 4.

To determine whether NO synthase is involved in the relaxation associated with 435 nm blue light treatments, we exposed colonic rings in organ bath *ex vivo* experiments to 100 μM L-NAME or buffer along with 28 μM of 9-cis retinal and 20 μM of GRK2 inhibitor {Methyl 5-[(E)-2-(5-nitrofuran-2-yl)ethenyl]furan-2-carboxylate} prior to 100 μM carbachol. In the buffer pretreatment group, 435 nm light treatment demonstrated a 32.8 ± 11.41% (mean ± SEM, *n* = 4), compared to the 100 μM L-NAME pretreatment group which demonstrated a 45.0 ± 7.35% (mean ± SEM, *n* = 4), during 435 nm blue light treatment. There was not a statistical difference between these two groups (Figure 6).

### Light-Induced Relaxation of Baseline Tone of Colonic Rings

**Figure 7:**
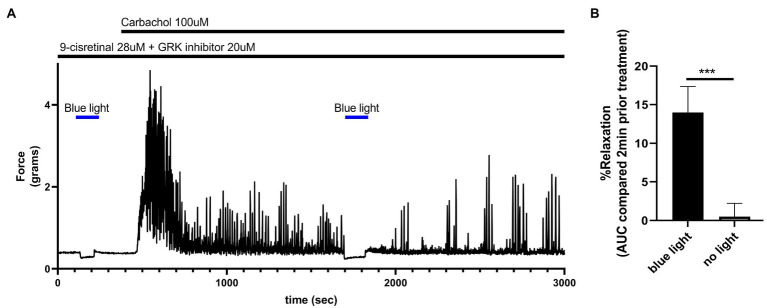
**(A)** Representative force tracing of colonic rings pretreated with 9-*cis* retinal 28 μM + GRK2 inhibitor 20 μM and constricted with 100 μM carbachol. Pretreatments depicted by the black bars. Blue bar depicts a 2 min 435 nm blue light treatment. Blue light treatment prior to constriction depicts the effects of blue light on resting tension. The second blue light treatment was to demonstrate the maintenance of post-contractile relaxation. **(B)** Area under the curve analysis of blue light-induced photorelaxation of rings at resting tension vs. colonic rings that did not get blue light treatment at resting tension. There was significant relaxation with light treatments compared to no light treatments (14 vs. 0.5%, respectively), *n* = 4, ^***^p < 0.001.

To determine the functional effects of light treatment on colonic tissue at rest, murine colonic rings in an organ bath were pretreated with 28 μM of 9-cis retinal and 20 μM GRK2 inhibitor {Methyl 5-[(E)-2-(5-nitrofuran-2-yl)ethenyl]furan-2-carboxylate}. About 435 nm blue light or no light treatment was performed for 2 min. Blue light induced a statistically significant (*p* < 0.001) relaxation from basal tone (14.0 ± 3.37%, mean ± SEM, *n* = 4) compared to the no light treatment controls (0.05 ± 1.73%, mean ± SEM, *n* = 4; Figure 7).

### KCl-Mediated Contraction of Murine Colon Has a Reduced Response to Photorelaxation

**Figure 8:**
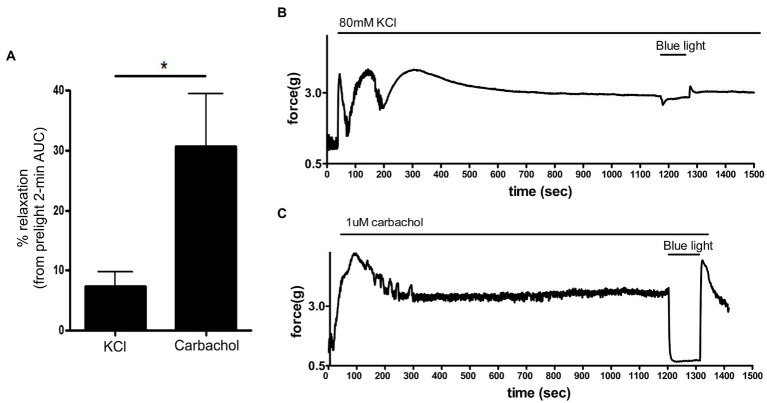
**(A)**: Area under the curve analysis of blue light-induced photorelaxation of rings precontracted with either carbachol 1 μM or KCl 80 mM. Rings precontracted with KCl exhibited significantly reduced photorelaxation in response to blue light (7.4 ± 2.3%) compared to rings precontracted with carbachol (31 ± 8.6%; ^*^p < 0.05, *n* = 8 rings per group). **(B,C)** Representative muscle force tracings of murine colon rings pretreated with TTX 1 μM, 9*-cis* retinal 28 μM + GRK2 inhibitor 20 μM and precontracted with either carbachol 1 μM or KCl 80 mM and subsequently exposed to blue light (435 nm λ) in wire myography experiments.

Exposure of smooth muscle to high external concentrations of potassium induces membrane depolarization and contraction that is independent of G protein-coupled receptor pathways. To determine whether photorelaxation of *ex vivo* murine colonic rings occurred following membrane depolarization, we pretreated dark-adapted colonic rings with the known opsin chromophore 9*-cis* retinal and GRK2 inhibitor, precontracted them with carbachol 1 μM or KCl 80 mM, and then exposed them to blue light (Figure 8). KCl-contracted rings demonstrated blue light-induced relaxation of 7.4 ± 2.3% (mean ± SEM, *n* = 8), while the carbachol-contracted rings demonstrated significantly greater blue light-induced relaxation of 30.8 ± 8.6%, (mean ± SEM, *n* = 8).

### CMMC Frequency Is Reduced by Blue Light

**Figure 9:**
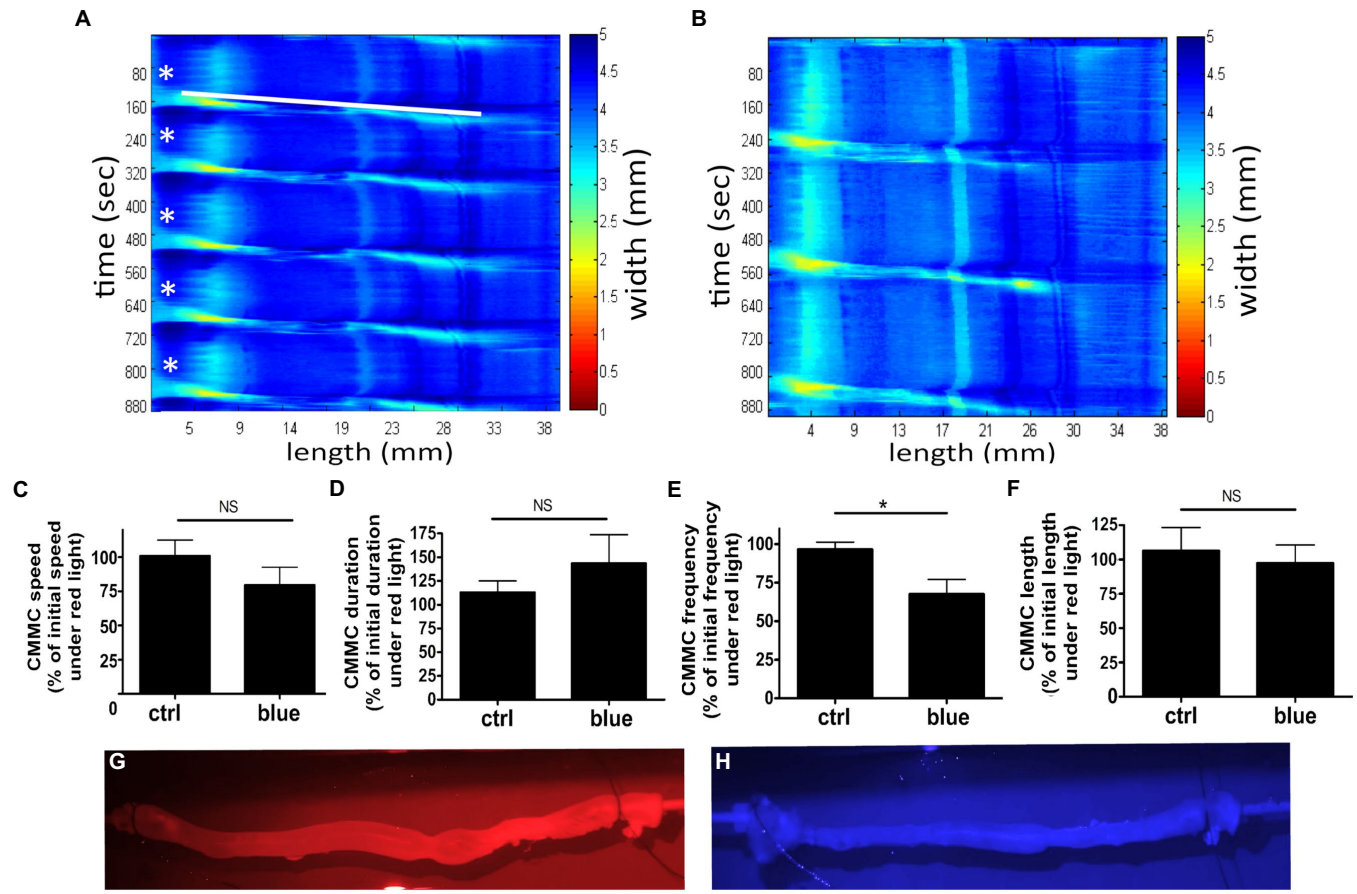
Murine colons were pretreated with 9*-cis* retinal 28 μM and were equilibrated under 660 nm λ (red) light and baseline motility recorded. Subsequent exposure to either 435 nm λ (blue) light or red light (time decay control, ctrl) was then recorded. **(A,B)** Representative heat map of ctrl colon **(A)** or blue light-treated colon **(B)**, using edge detection during spontaneous colonic peristalsis. Heat map displays time on the y-axis and length along the colon on the x-axis. Color changes in the heat map indicate a change in the width of the colon at the correlating length and correlating time on the x and y-axes. The start of each colonic migrating motor complexes (CMMC) is labeled in **(A)** with a white star, and the progression of the first CMMC down the length of the colon is depicted by the white bar. **(C–F)** Graphical representation of measured speed, duration, frequency, and length of recorded colonic motility. Ctrl represents colons recorded under red light as a percentage of their baseline red recording as a time decay control. Blue represents blue light-treated colons as a percentage of their baseline red recording. **(E)** Frequency of CMMC decreased more with blue light exposure to 68 ± 9.4% of baseline, while ctrl decreased to 97 ± 4.6% of baseline (^*^p < 0.05, *n* = 5–9). **(G,H)** Captured frames of video recording colonic motility during red and blue light exposures demonstrating the captured visual quality under each lighting condition.

Colonic migrating motor complexes are neurogenic motor behaviors of the colon that involve the enteric nervous system (ENS) and all the cellular constituents of the smooth muscle syncytium including the interstitial cells of Cajal (ICCs), PGFRα-expressing cells, and smooth muscle cells (Bayguinov et al., 2010a; Smith and Koh, 2017). Luminal distension of the gut is directly and indirectly detected by afferent neurons in the myenteric plexus to trigger activation and contraction of the smooth muscle layers in a cyclical pattern (Smith et al., 2014). This cyclical pattern is a summation of activation and release of tonic inhibitory signals and is influenced by extrinsic nerves as well as epithelial cells (Li et al., 2002; Drumm et al., 2019). To determine the functional effect of photorelaxation on the cyclical peristaltic movement of the colon and the colonic motor complex (nerves, myenteric plexus, smooth muscle, and epithelial cells), *ex vivo* intact murine colons were pretreated with 9*-cis* retinal, primed with a small amount of luminal hydrostatic pressure (distension), and exposed to blue light (Figure 9). The setup and initial (baseline) video imaging were performed under red light, and subsequent recordings were made either under continued red light exposure or under blue light treatment. Speed, length, duration, and frequency of CMMC were analyzed as percent of baseline values. When compared to the red light-only treated time decay control colons, blue light-treated colons demonstrated a significant difference in CMMC frequency alone. The time decay control group (red light only) demonstrated 96.7 ± 4.6% (mean ± SEM, *n* = 5) of its baseline frequency, while blue light treatment group demonstrated 67.6 ± 9.4% (mean ± SEM, *n* = 9) of its baseline frequency (*p* < 0.05). Blue light-treated colons did not significantly change with regard to CMMC speed, duration, or length compared to the control group.

## Discussion

In the present study, we show that blue light exposure relaxes precontracted colonic tissue segments and reduces the frequency of spontaneous colonic motor contractions in mouse colons *ex vivo*, and we implicate opsin photoreceptors as mediators of this effect. We demonstrate that 9-cis retinal, a derivative of the classical endogenous ligand for opsin, sensitizes tissues to light-mediated relaxation in the blue spectrum. We further implicate a GPCR pathway by demonstrating augmentation of the 9-cis retinal sensitization by inhibition of a known GPCR desensitization pathway; G protein receptor kinase. We further characterize the photorelaxation and demonstrate persistent relaxation in the presence of L-NAME, a potent NOS inhibitor, as well as tetrodotoxin, an inhibitor of sodium channels on many types of neurons.

Opsin expression and smooth muscle functional modulation *via* opsins have been studied in other organs (Sikka et al., 2014; Barreto Ortiz et al., 2018; Yim et al., 2019, 2020). Here, we confirmed expression of the OPN3 receptor protein in mouse colon, localizing to a subset of neurons within the myenteric plexus. OPN3 has previously been shown to function as a photoreceptor in human colon cancer cells (Yoshimoto et al., 2018). Zebrafish intestine has been shown to express opsins (Davies et al., 2015) and to exhibit light-entrainable circadian clocks (Pardini et al., 2005; Li et al., 2008; Peyric et al., 2013). As hypothesized by others, endogenous gut opsin may play a role in syncing vertebrate digestive processes with light/dark cycles (Davies et al., 2015).

There are estimated to be at least 14 distinct types of neurons in the colonic myenteric plexus (Drokhlyansky et al., 2020; May-Zhang et al., 2021), many of which have a role in regulating gut motility including intrinsic primary afferent neurons (IPANs), inhibitory motor neurons, excitatory motor neurons, and interneurons (Furness et al., 1998; Bayguinov et al., 2010b; Hennig et al., 2015). These neurons release both procontractile factors (serotonin, acetylcholine, neurokinins, and cholecystokinin) and prorelaxant factors (vasoactive intestinal peptide and nitric oxide; Barone et al., 1989; Plujà et al., 2000; Li et al., 2002; Spencer, 2013; Smith and Koh, 2017). The presented CMMC experiments demonstrate the organ effects of blue light on spontaneous tension generation and signal propagation of colonic contractions. CMMC frequency was significantly decreased in the presence of blue light treatment, while all other parameters of speed, length, and duration of peristalsis were unchanged. This suggests that OPN3-expressing neurons have a role in setting the frequency of colonic contractions and this regulation is modulated by light exposure. Serotonergic signaling from enterochromaffin cells to IPANs has an important role in colonic motility, and 5HT3R inhibition with ondansetron is known to decrease CMMC frequency and alter other CMMC properties (Heredia et al., 2013; Spencer et al., 2013). Since blue light only reduced the frequency of CMMCs, this may be an indication that IPANs are the key OPN3-expressing neurons in the ENS. Major inhibitory neuronal inputs have been described as exerting tonic inhibitory effect on smooth muscle *via* the neuronal release of NO to the circular and longitudinal muscle layers (Spencer, 2013). In our *ex vivo* study, however, pretreatment of colonic rings with a potent inhibitor of nitric oxide synthase, L-NAME, did not alter blue light-mediated relaxation. This suggests that NOS-mediated pathways are not a primary mechanism of blue light-mediated relaxation. At this point, deciphering which neuronal pathway solely influences the frequency would be highly speculative and more than one cell type may be responsible for the decrease in CMMC frequency.

While whole colon motility was studied without nerve blockade, some of the force measurement studies were performed with TTX. TTX pretreatments would exclude the majority of fast synaptic transmission in the ENS but does not exclude possible contributions of TTX-insensitive cells. Neuronal expression of Nav1.9, which is TTX-insensitive, has been demonstrated in colonic neurons including IPANs (Rugiero et al., 2003; Mao et al., 2006). IPAN is known to have an extended hyperpolarization after an action potential *via* calcium-activated potassium currents (KCa; Vogalis et al., 2001). Light may affect these neurons by augmenting the KCa activity through G protein-coupled receptors (as seen in beta receptors). Altered Nav1.9 expression in mice decreased the frequency of spontaneous CMMC (Copel et al., 2013). Non-neuronal cells also demonstrate sodium currents that are resistant to tetrodotoxin (smooth muscle cells, ICC, and glial cells; Strege et al., 2003). Interstitial cells of Cajal transmit a tetrodotoxin-insensitive signal to smooth muscles (both circular and longitudinal) *via* gap junctions (Sarna, 2008) and in the form of inward and outward currents (Gomez-Pinilla et al., 2009; Zhu et al., 2009). Colonic smooth muscle cells also express sodium channels that are insensitive to tetrodotoxin and possibly are modulated to effect CMMC frequency. According to a colonic smooth muscle transcriptome analysis, Opsin 3 mRNA was identified in both ICC and colonic smooth muscle, albeit in varying amounts (ICC more than smooth muscle; Ha et al., 2017). Lastly, glial cells have both TTX sensitive and insensitive functions and demonstrated an effect on CMMC frequency without other changes in duration or speed (Rao et al., 2017).

The eGFP expression (Figure 3) demonstrates distinct staining in the neuronal plexus of the colonic wall, while the staining within the muscle layer (ICC, smooth muscle) is dramatically less. Opsin 3 activation on muscle/interstitial cells may explain the *ex vivo* colonic ring studies demonstrating persistent photorelaxation in the presence of tetrodotoxin. It is possible that the tethered eGFP (33 kDa), a relatively large molecule, may hinder the expression of Opsin 3 to the plasma membrane of smooth muscle or ICC cells during post-translational regulation. It is possible that the eGFP signal does not accurately demonstrate endogenous expression in certain cell types. While expression of opsins on colonic neurons can more accurately explain the inhibitory effects of light on CMMC, it is not as obvious how neurons can relax directly stimulated colonic contraction in the *ex vivo* murine colonic ring organ bath studies. We expand our CMMC hypothesis that light may be inhibiting neuronal firing of stretch/tone-induced IPAN. Basal tone created by elongation was calibrated to 1 g of isometric tension in each colonic ring during *ex vivo* murine colonic ring organ bath experiment. This basal tone could possibly elicit activation of IPANs (Kunze et al., 1998), which we hypothesize may have been inhibited by blue light exposure (Figure 7). IPANs which express NaV 1.9 (Vogalis et al., 2001), tetrodotoxin-insensitive channel, would then increase in firing during carbachol treatments due to smooth muscle constriction (Kunze et al., 1999). Carbachol-induced increase in colonic tone was inhibited by blue light exposure, possibly inhibiting IPAN activation during constriction (Figure 7). IPAN canonically interacts with neurons that are typically sensitive to tetrodotoxin to effect smooth muscle tone. Thus, this hypothesis suggests that IPANs are directly interacting with ICC/smooth muscle or affect other tetrodotoxin-insensitive colonic neurons.

Although the effects of blue light on each colonic cell type are not clearly defined, the cumulative effect of light on whole murine colon is a definitive decrease in the frequency of colonic motility. Blue light may be inhibiting the effects of neurons, relaxing smooth muscle, deactivating ICCs, or a combination of these effects. Glia and enteroendocrine cells may also be influenced by blue light exposure, indirectly. The current study demonstrates expression of Opsin 3-eGFP-tagged protein in neurons within the myenteric plexus as well as low-level staining within the smooth muscle layer. Further exploration into the colonic cell types that express Opsin 3 is needed as well as studies to correlate the expression and function of human opsins to our current mouse studies.

Retinal derivatives are needed to be covalently bound to opsins in order to form a photosensitive retinylidene. When constituted with the chromophore 11*-cis* retinal, the maximum absorption spectrum of dark-adapted, vertebrate OPN3 is ~470 nm λ (Koyanagi et al., 2013; Sugihara et al., 2016). In the present study, we used shorter wavelength blue light, because we performed a wavelength scan in our previous report on OPN3 activation in airway smooth muscle and determined that 440 nm λ light produced maximal relaxation (Yim et al., 2019). The apparent blue shift in OPN3 absorption can be explained by the switch to 9*-cis* retinal as a chromophore. OPN3 apoprotein constitution with different chromophores can either red- or blue-shift the photopigment’s absorption spectrum, and the use of 9*-cis* retinal has been shown to exhibit blue-shifted maxima relative to 11*-cis* retinal-OPN3 (Hubbard and Wald, 1952; Koyanagi et al., 2013). With the use of the 9-*cis* retinal, we are able to demonstrate the ligand-dependent light sensitivity at the ~440 nm wavelength. Studies utilizing OPN3 null animals as well as tissue-specific OPN3 genetic deletions will further elucidate the role of OPN3 in photorelaxation and retinal sensitization. Differences in the magnitude and duration of colonic smooth muscle relaxation in studies using 9-cis retinal presented in Figures 1, 2 were observed. These differences can be explained by three factors: (1) animal differences (within one experiment we use colonic rings from the same colon in order to decrease variability between animals), (2) pretreatment times of 9-cis retinal were all 1 h prior to light treatment in Figure 1 it was only 15 min prior to light treatment (longer treatment times lead to greater tissue penetrance), and (3) there were two blue light exposures of tissues in Figure 1 prior to the 28 μM 9-cis retinal treatment possibly desensitizing the receptor (We demonstrate the enhancement of the light effect when desensitization pathways are blocked with a GRK2 inhibitor.)

Since the wavelengths we used in our experiments (λ < 450 nm), are close to the ultraviolet spectrum, we were concerned that some of the photorelaxation we observed is due to UV A (UVA)-induced NO production. UV-induced release of endothelial NO has been shown to have vasodilatory effects (Holliman et al., 2017), and UV light (wavelengths considerably smaller than 435 nm) has been used to reduce the basal tone of rat duodenum through a suspected NO mechanism (Dave et al., 1979). However, UVA light occurs at wavelengths considerably shorter than what was used in these experiments, 435 nm. Furthermore, a NO-dependent mechanism is not consistent with the colonic photorelaxation, we observed with 9*-cis* retinaldehyde pretreatment: To the contrary, retinoids have been shown to inhibit NO synthesis in smooth muscle (Hirokawa et al., 1994; Sirsjö et al., 2000) *via* NOS mRNA. While it is possible that oxidation of 9*-cis* retinal by enteric cells may produce retinoids which in turn affects transcription by binding to nuclear retinoic acid receptors (Wright-Jin et al., 2013), significant changes in protein expression due to changes in retinoic acid levels take at least several hours to occur (Balmer and Blomhoff, 2002; Paradkar and Roth, 2006). As none of our experiments in which 9*-cis* retinal was used exceeded 1 h of retinal pretreatment before blue light exposure, it is unlikely that genomic, transcriptional events increased smooth muscle photosensitivity. Instead, we believe 9*-cis* retinal’s enhancement of blue light’s antispasmodic effect to be due to 9*-cis* retinal’s serving as a chromophore ligand to the OPN3 receptor. However, in some of our organ bath experiments where we measured tension of the colonic rings without the addition of GRK inhibitor, we did notice a transient relaxation in both low concentrations of retinal and vehicle-treated groups. We hypothesize that this initial transient relaxation might be due to photoisomerization of pre-existing, opsin-bound chromophores, which we hypothesize to quickly to be depleted and/or desensitized.

In our studies, GRK2 inhibition augmented the 9-*cis* response to light and further bolsters a GPCR-mediated mechanism of relaxation. Phosphorylation of GPCRs by GRKs leads to β-arrestin binding and receptor endocytosis (Moore et al., 2007). The involvement of this GPCR kinase-mediated, negative feedback mechanism is consistent with a previous study of opsin desensitization mechanisms (Shichi and Somers, 1978). Furthermore, the specific involvement of GRK2, which classically mediates desensitization of β-adrenoceptors that signal through the G_s_α pathway, suggests that colonic opsin may also couple with G_s_α (Lohse et al., 1992). The enhanced relaxation *via* the addition of GRK2 inhibitor is consistent with a decrease in desensitization of G protein-coupled receptors, concluding that the effects of blue light on neurons or a downstream prorelaxant target are G protein mediated. Vertebrate OPN3 has been shown to couple with G_i_α and G_o_α, and human epidermal OPN3 functions through G_i_α (Koyanagi et al., 2013; Ozdeslik et al., 2019). In smooth muscle, G_i_α signaling counteracts the G_s_α pathway and contributes to calcium sensitization (Croxton et al., 1998; Billington and Penn, 2003). In contrast, smooth muscle OPN3 has been associated with G_s_α by coimmunoprecipitation and proximity ligation assay results in previous studies conducted in human myometrium and airway (Yim et al., 2020; Wu et al., 2021). OPN3’s G protein specificity appears to vary not only between species, but also between tissue/cell types.

To further investigate the cellular mechanisms of blue light-mediated photorelaxation, we questioned the ability of blue light to relax smooth muscle contraction induced by membrane depolarization. Increasing the external concentration of K^+^ decreases outward K^+^ current by changing the equilibrium potential of K^+^ (Soder and Petkov, 2011). Blue light had reduced spasmolytic effect on colonic rings precontracted with KCl compared to rings precontracted to the same magnitude with carbachol. We conclude that blue light mechanism of relaxation is upstream from convergent downstream mechanisms shared by KCl and carbachol constrictions (e.g., actin–myosin cross-linking; Mangel, 1984; Nasu et al., 1995; Otto et al., 1996).

Non-visual, extraocular opsin photoreceptors were first discovered in 1994 in chicken pineal glands (Okano et al., 1994). Still, the function of opsins outside the central nervous system, especially in internal organs, remains poorly understood (Terakita, 2005). Although there is controversy that visible light with wavelengths shorter than red light can penetrate deeper than a few millimeters into tissue (Ash et al., 2017), the existence of an endogenous ligand capable of activating non-visual opsins in humans has not be ruled out (Kefalov et al., 1999; Isayama et al., 2009). These ligands are important, and specificity can vary between different opsin subtypes. Channel rhodopsin, typically used in optogenetics, uses all-trans retinal to increase their light sensitivity (Kleinlogel et al., 2011). Opsin 3, however, uses a *cis* form of retinal to increase it sensitivity and may be necessary for any activity of Opsin 3. Targeting of opsin photoreceptors for the sake of treating diseases such as IBS may not rely solely on the delivery of photons. IBS pathogenesis is complicated and has been associated with inflammatory, neuronal, psychological, and, more recently, microbial factors (Lovell and Ford, 2012; Geng et al., 2018; Chong et al., 2019). Opsin 3 has been found in a multitude of tissues, and its role in contraction relaxation is not fully understood. The potential to modulate colonic motor behaviors with light has therapeutic potential to help IBS patients with colonic dysmotility.

## Data Availability Statement

The raw data supporting the conclusions of this article will be made available by the authors upon request, without undue reservation.

## Ethics Statement

The animal study was reviewed and approved by Columbia University Institutional Animal Care and Use Committee.

## Author Contributions

WD, GP, ADW, MR, RAL, and PDY contributed to study design. WD, GP, ADW, SV, and PDY performed the experiments. WD, GP, ADW, KP, MR, SV, and PDY analyzed and interpreted the data. WD, GP, SV, and PDY wrote the manuscript and prepared figures. WD, GP, ADW, KP, MR, DEB, RAL, and PDY revised and approved the manuscript before submission. All authors contributed to the article and approved the submitted version.

## Funding

This work was supported by National Institutes of Health grants 1K08HL150314-01A1 (PDY), 5K08DK110532-06 (MR), R01EY032029 (RAL), and R01EY032752 (RAL).

## Conflict of Interest

The authors declare that the research was conducted in the absence of any commercial or financial relationships that could be construed as a potential conflict of interest.

## Publisher’s Note

All claims expressed in this article are solely those of the authors and do not necessarily represent those of their affiliated organizations, or those of the publisher, the editors and the reviewers. Any product that may be evaluated in this article, or claim that may be made by its manufacturer, is not guaranteed or endorsed by the publisher.
